# Supplementary material for: Evaluation and comparison of scoring systems for predicting stone-free status after flexible ureteroscopy for renal and ureteral stones
Source: PLoS One. 2020 Aug 6;15(8):e0237068. doi: 10.1371/journal.pone.0237068 (PMC7410314; doi:10.1371/journal.pone.0237068)
Supplement: Attachment — Submitted filename: Responses to reviewers.docx [file pone.0237068.s002.docx]

Pr François KLEINCLAUSS

Department of Urology and Renal Transplantation

University Hospital of Besançon

3 boulevard Alexandre Fleming

25000 BESANCON

FRANCE

[francois.kleinclauss@univ-fcomte.fr](mailto:francois.kleinclauss@univ-fcomte.fr)

Dr. Joerg HEBER

Editor in Chief, PLOS ONE

April 20, 2020

Dear Dr. Joerg HEBER,

We thank the Editorial Board and the reviewers for their critical and empathic assessment of our work. Their comments allowed us to improve the quality of our work. In the following letter we address a point by point answer to their comments.

We hope that the Editorial Board as well as the expert reviewers will find our manuscript of interest for the readership of *Plos One*.

With my best regards,

Pr. François Kleinclauss, (MD, PhD)

**Point by point answer**

Journal requirements:

*When submitting your revision, we need you to address these additional requirements:*

*1.    Please ensure that your manuscript meets PLOS ONE's style requirements, including those for file naming.*

The file naming have been changed

*2. In ethics statement in the manuscript and in the online submission form, please provide additional information about the patient records/samples used in your retrospective study. Specifically, please ensure that you have discussed whether all data/samples were fully anonymized before you accessed them and/or whether the IRB or ethics committee waived the requirement for informed consent. If patients provided informed written consent to have data/samples from their medical records used in research, please include this information.'*

We Provide additional information in the online submission.

All data were anonymized

Ethics committee waived the requirement for informed consent.

The « Délégation a la Recherche Clinique et à l’innovation » approved the study. This institution is certified ISO9001v15.

3. *PLOS requires an ORCID iD for the corresponding author in Editorial Manager on papers submitted after December 6th, 2016. Please ensure that you have an ORCID iD and that it is validated in Editorial Manager. To do this, go to ‘Update my Information’ (in the upper left-hand corner of the main menu), and click on the Fetch/Validate link next to the ORCID field. This will take you to the ORCID site and allow you to create a new iD or authenticate a pre-existing iD in Editorial Manager.*

We update this information.

ORCID iD for the corresponding author is : 0000-0002-1049-0117

*4. Thank you for stating the following financial disclosure: "no"*

*At this time, please address the following queries:*

*a)    Please clarify the sources of funding (financial or material support) for your study. List the grants or organizations that supported your study, including funding received from your institution.*

*b)    State what role the funders took in the study. If the funders had no role in your study, please state: “The funders had no role in study design, data collection and analysis, decision to publish, or preparation of the manuscript.”*

*c)     If any authors received a salary from any of your funders, please state which authors and which funders.*

*d)     If you did not receive any funding for this study, please state: “The authors received no specific funding for this work.”*

 The authors received no specific funding for this work. The authors didn’t receive a salary.

*Please include your amended statements within your cover letter; we will change the online submission form on your behalf.*

The cover letter has been modified.

*5. Thank you for stating the following in your Competing Interests section:  "no"*

*Please complete your Competing Interests on the online submission form to state any Competing Interests. If you have no competing interests, please state "The authors have declared that no competing interests exist.", as detailed online in our guide for authors at* [*http://journals.plos.org/plosone/s/submit-now*](about:blank)

*This information should be included in your cover letter; we will change the online submission form on your behalf.*

 The cover letter has been modified.

“We have no conflicts of interest to disclose.” change to “The authors have declared that no competing interests exist.” in the cover letter.

*Please know it is PLOS ONE policy for corresponding authors to declare, on behalf of all authors, all potential competing interests for the purposes of transparency. PLOS defines a competing interest as anything that interferes with, or could reasonably be perceived as interfering with, the full and objective presentation, peer review, editorial decision-making, or publication of research or non-research articles submitted to one of the journals. Competing interests can be financial or non-financial, professional, or personal. Competing interests can arise in relationship to an organization or another person. Please follow this link to our website for more details on competing interests:*[*http://journals.plos.org/plosone/s/competing-interests*](about:blank)

*Reviewers' comments:

Reviewer's Responses to Questions*

*Reviewer #1: The study aims to compare four previously developed predictive scores for stone-free rate (SFR) after flexible ureterorenoscopy (f-URS) with holmium-YAG laser fragmentation of renal and ureteral lithiasis. The study was single-center and used between 669-800 procedures to evaluate the four predictive scores. Two scores have been previously validated- the Resolu Unsal Stone Score (RUSS) and modified Seoul National University Renal Stone Complexity (S-ReSC) score. The other two scores, the S.T.O.N.E score described by Molina et al. and Ito’s nomogram have not been validated in any other cohorts.

The study has a clear objective, the end point is concretely defined, and the analysis is straightforward. However, the generalizability of the findings is unclear especially since this is a single center study with fewer than 1000 cases and the use of a single rater. Overall, this manuscript sets the stage for future studies that can further validate the clinical utility of the predictive scores.*

*Major points:*

*1) Inclusion of the absolute numbers of patients would be informative for Tables 1 and 2. For example, how many patients are in UR and RO groups for Table 1? How many patients are in the Yes and No groups for Table 2?*

We added the number of patients and the number of procedures in Table 1 : “Number of patients : 577 for UR group and 484 patients for RO group”

Nevertheless, each procedure was considered on an individual basis.

In Table 2, we added the number of procedures in each group.

*2) It is very difficult for this reader to decipher information in Table 2. It is difficult to calculate the percentages by how the authors display the numbers. The authors describe the proportion of patients by no/yes and %yes. I believe it is easier to interpret the percentages if the authors present the fraction of patients who were yes/total patients. For example, the pre-stenting group is show as "207/385". It should be changed to 385/592 or 65% so that the reader can see more easily for how the percentages were calculated. This should be done for all of the fractions in Tables 2 and 3.*

The suggested modifications have been made for Tables 2 and 3.

*3) Table 3: STONE score section is very difficult to interpret. What do the numbers represent? I believe that it is unrealistic to expect the reader to be familiar with the STONE score section without an explanation for what the scores mean. Some context in the manuscript would be helpful so that the reader can understand the basics of the STONE score system.*

The STONE score and the other scores are defined in the Material and Method section. The scoring system has been added in the table 3. We add line 103 “(5 to 15 points). A high total score is predictive of a failure f-URS outcome.”

*4) Table 3 has error in reporting the data
Lower pole location- %yes = 85.7%, not 14.3% (yes group)
Lower pole location- %yes = 75.9%, not 24.1% (no group)*

We checked all the data. After verification there was a little error in these data. However, these numbers correspond to Lower pole location associated with infendibulo-pelvic angle < 45°.

71/482 (14,7%) of the procedures includes these parameters for ‘Yes stone free groupe’

43/187 (23.0%) of the procedures includes these parameters for ‘No stone free groupe’

*Stone number in different calyces > 1 - %yes = 22.8%, not 37.4% (yes group)*

The suggested modifications have been made.
*Stone number in different calyces > 1 - %yes = 37.4%, not 22.8% (no group)*

The suggested modifications have been made.

*Hydronephrosis- %absent = 61%, not 39% (yes group)
Hydronephrosis- %absent = 60.4%, not 39.6% (no group)*

The suggested modifications have been made. We reported the percentage of absent hydronephrosis because in this score, the absence of hydronephrosis is quoted.

Similarly, “lower pole calculi” change to “No lower pole calculi” in Ito’s score item, for easier interpretation.

*5) For a non-urology audience it is unclear what role a predictive score for SFR would play in clinical decision making. It would be helpful to address the following in the discussion: if there was a perfect predictive score that was 100% sensitive and specific, would a particular score keep a physician from intervening with f-URS or rather is the score meant to be helpful for patient counseling and managing patient expectations. Explicitly stating the clinical impact of using a predictive score would be useful context.*

We completely agree with this comment and add the following sentences in the discussion section line 254 :

“Based on our results it’s difficult to rank this different scores, according there accuracy. We found that three factors i.e. stone size, stone number, lower pole and IPA < 45° mainly impact the scores results. Moreover, the remaining question is the clinical impact of such scores. Independently of the information gave to patients and maybe to help the choice of surgical technique this kind of scores are not clinically very usefull and are not routinely used for many reasons (difficulty and time to calculate). The RUSS score needs IPA angle definition on CT scan, the Ito’s score required the use of nomogram and the measurement of the stone volume, the STONE score can be calculated mentally in daily practice and the Seoul score would be the more easy to use.”

Answer to Reviewer #2 :

*The authors evaluated four predictive scoring models for stone-free rate after flexible ureteroscopic urinary stone surgery. Major pitfall of the study is lack of systemic and consistent post-operative imaging to evaluate stone-free. I have some other concerns and questions.*

We agree with this comment and the absence of systematic post-operative imaging is one of the major bias of our study. This bias is reported in the discussion section. About a half of the patients underwent post-operative imaging but all underwent endoscopic and fluoroscopic inspection after surgery.

*1. Title: I think all of them are not nomograms, but they are predictive model or scoring system. Thus, the title should be revised with appropriate term.*

We completely agree with this comment.

The title was changed for “ **Evaluation and comparison of scoring systems for predicting stone-free status after flexible ureteroscopy for renal and ureteral stones”**

*2. Stone composition analysis is importance information when interpreting results of urinary stone surgery. Please, provide the results (at lease, proportion of uric acid stone)*

We add these data in the result section and reported the repartition of the stone composition. Line 146 :

“Repartition of chemical composition : 66 % were calcium oxalate, 14% were calcium phosphate, 9% were uriq acid and 11% were other.”

*3. How many surgeons involved the study? Didn't they have different patient selection or surgical outcomes? How did you adjust for surgeon effect?
Please, additionally provide brief description of their experience.*

We add the number of surgeons line 237 : (12 operators)

These procedures were performed by residents, novices and experienced surgeons. This reflects real-life urological practice. The impact of experience and learning curve were not the goal in this paper but are the topics of another paper currently in writing. Moreover, we previouly reported that surgeon with less than 20 f-URS had similar results than surgeon with more than 20 f-URS procedures (Kleinclauss F, EAU2013)

4*. The major advantage of the study is concurrent comparison of 4 existing predictive models to predict stone-free after flexible ureteroscopic stone surgery. If you discuss it in practical aspect with balanced manner, not only focusing to the predictive ability, it can be very valuable discussion. For example, granularity of score or how many factors do they need can effect the predictive ability. By contrast, how difficult to calculate the score and how much time do they need can diminish the ability and clinical usefulness.*

We agree with this comment and add a paragraph in the Discussion section about the routine use of such score (line 254) :

“Based on our results it’s difficult to rank this different scores, according there accuracy. We found that three factors i.e. stone size, stone number, lower pole and IPA < 45° mainly impact the scores results. Moreover, the remaining question is the clinical impact of such scores. Independently of the information gave to patients and maybe to help the choice of surgical technique this kind of scores are not clinically very usefull and are not routinely used for many reasons (difficulty and time to calculate). The RUSS score needs IPA angle definition on CT scan, the Ito’s score required the use of nomogram and the measurement of the stone volume, the STONE score can be calculated mentally in daily practice and the Seoul score would be the more easy to use.”
