# Supplementary material for: Evaluation and comparison of scoring systems for predicting stone-free status after flexible ureteroscopy for renal and ureteral stones
Source: PLoS One. 2020 Aug 6;15(8):e0237068. doi: 10.1371/journal.pone.0237068 (PMC7410314; doi:10.1371/journal.pone.0237068)
Supplement: Attachment — Submitted filename: Responses to reviewers .docx [file pone.0237068.s003.docx]

Pr François KLEINCLAUSS

Department of Urology and Renal Transplantation

University Hospital of Besançon

3 boulevard Alexandre Fleming

25000 BESANCON

FRANCE

[francois.kleinclauss@univ-fcomte.fr](mailto:francois.kleinclauss@univ-fcomte.fr)

Dr. Joerg HEBER

Editor in Chief, PLOS ONE

April 20, 2020

Dear Dr. Joerg HEBER,

We thank the Editorial Board and the reviewers for their critical and empathic assessment of our work. Their comments allowed us to improve the quality of our work. In the following letter we address a point by point answer to their comments.

We hope that the Editorial Board as well as the expert reviewers will find our manuscript of interest for the readership of *Plos One*.

With my best regards,

Pr. François Kleinclauss, (MD, PhD)

**Point by point answer :**

Reviewers' comments:

6. Review Comments to the Author

Please use the space provided to explain your answers to the questions above. You may also include additional comments for the author, including concerns about dual publication, research ethics, or publication ethics. (Please upload your review as an attachment if it exceeds 20,000 characters)

Reviewer #1: The following comments were adequately addressed:

1. The authors have added UR and RO patient numbers to Tables 1 and 2 and now provide helpful context.
2. The authors have provided fractions in Table 2 and now the data presentation is more clear.
3. The authors have provided a range for the S.C.OR.E system and they now provide an explanation for the meaning of a high score in line 103.


Minor Revisions:

1. We request that the authors take the time to make sure that the writing is clear and that grammar is used correctly. For example, there is a passage in the Discussion section that reads (starting line 259):

"Independently of the information gave to patients and maybe to help the choice of surgical technique this kind of scores are not clinically very usefull and are not routinely used for many reasons (difficulty and time to calculate)."

It is not clear to me what the authors are trying to say here. Please also spell check the document.

The suggested modifications have been made. We change this passage to “ Based on our results it is difficult to rank the different scores according their accuracy. We found that four main factors: stone size, stone number, lower pole and IPA <45° affected the scores. Moreover, we should also ask what the clinical impact of these scores is. Independent of the information given to patients and possibly to help in the choice of surgical technique, these kind of scores are not clinically useful and are not used routinely due to their difficulty and the time required to calculate them.”

We also spell check the document.

Starting line 261: "The RUSS score needs IPA angle definition on CT scan, the Ito’s score required the use of nomogram and the measurement of the stone volume, the STONE score can be calculated mentally in daily practice and the Seoul score would be the more easy to use."

There are 5 sentences here that are connected together into 1 large sentence. Please make this sentence more clear to the reader, perhaps the authors should break up the sentence into smaller sentences.

The suggested modifications have been made. We change this sentence to : “The RUSS score needs IPA angle definition on a computed tomography scan and the Ito score requires the use of a nomogram and measurement of the stone volume. Conversely, the S.T.O.N.E. score can be calculated mentally in daily practice and the Seoul score would be easiest to use.”


2. Please make uniform use of the decimal point or the comma. Please be consistent with the usage according to PLOS-ONE guidelines. For example:

Table 2, Lines 1 and 2 should read "25.9" and "27.9" as opposed to "25,9" and "27,9."

The suggested modifications have been made.

Table 3, missing a paranthesis before "14.7)" and should read "36.1" as opposed to "36,1".

The suggested modifications have been made.

3. In the Discussion section, there are three 1-sentence paragraphs and two 2-3 sentence paragraphs. In general, paragraphs should contain a collection of sentences that convey a related thought or message. The Discussion section could benefit from fewer paragraphs that are longer, rather than several 1-2 sentence paragraphs.

The suggested modifications have been made.

Some paragraphs have been grouped.

Reviewer #2: Thanks for the appropriate revision.

One minor thing should be corrected before publication; "uriq acid" --> "uric acid".

This modification have been made.

[NOTE: If reviewer comments were submitted as an attachment file, they will be attached to this email and accessible via the submission site. Please log into your account, locate the manuscript record, and check for the action link "View Attachments". If this link does not appear, there are no attachment files.]

While revising your submission, please upload your figure files to the Preflight Analysis and Conversion Engine (PACE) digital diagnostic tool, [https://pacev2.apexcovantage.com/](https://eur03.safelinks.protection.outlook.com/?url=https%3A%2F%2Fpacev2.apexcovantage.com%2F&data=02%7C01%7C%7C76464d5b334647f4463808d81a89106d%7C84df9e7fe9f640afb435aaaaaaaaaaaa%7C1%7C0%7C637288523271198830&sdata=PKxuK%2Foz7pJT%2BNXaHZpRMXHDUYZY%2FI4wW1pZDjGqBgU%3D&reserved=0). PACE helps ensure that figures meet PLOS requirements. To use PACE, you must first register as a user. Registration is free. Then, login and navigate to the UPLOAD tab, where you will find detailed instructions on how to use the tool. If you encounter any issues or have any questions when using PACE, please email PLOS at [figures@plos.org](mailto:figures@plos.org). Please note that Supporting Information files do not need this step.

The figure files have been upload to the Preflight Analysis and Conversion Engine (PACE) digital diagnostic tool.
